# Transcriptional and functional predictors of potato virus Y-induced tuber necrosis in potato (*Solanum tuberosum*)

**DOI:** 10.3389/fpls.2024.1369846

**Published:** 2024-04-04

**Authors:** Richard Manasseh, Vidyasagar Sathuvalli, Hanu R. Pappu

**Affiliations:** ^1^ Department of Plant Pathology, Washington State University, Pullman, WA, United States; ^2^ Hermiston Agricultural Research and Extension Center, Oregon State University, Hermiston, OR, United States

**Keywords:** potato, potato virus Y, RNA-Seq, bioinformatics, GO terms, KEGG pathways, host response, plant-virus interactions

## Abstract

**Introduction:**

Potato (*Solanum tuberosum* L.), the fourth most important food crop in the world, is affected by several viral pathogens with potato virus Y (PVY) having the greatest economic impact. At least nine biologically distinct variants of PVY are known to infect potato. These include the relatively new recombinant types named PVY-NTN and PVYN-Wi, which induce tuber necrosis in susceptible cultivars. To date, the molecular plant-virus interactions underlying this pathogenicity have not been fully characterized. We hypothesized that this necrotic behavior is supported by transcriptional and functional signatures that are unique to PVY-NTN and PVYN-Wi.

**Methods:**

To test this hypothesis, transcriptional responses of cv. Russet Burbank, a PVY susceptible cultivar, to three PVY strains PVY-O, PVY-NTN, and PVYN-Wi were studied using mRNA-Seq. A haploid-resolved genome assembly for tetraploid potato was used for bioinformatics analysis.

**Results:**

The study revealed 36 GO terms and nine KEGG 24 pathways that overlapped across the three PVY strains, making them generic features of PVY susceptibility in potato. Ten GO terms and three KEGG pathways enriched for PVY-NTN and PVYN-Wi only, which made them candidate functional signatures associated with PVY-induced tuber necrosis in potato. In addition, five other pathways were enriched for PVYNTN or PVYN-Wi. One carbon pool by folate was enriched exclusively in response to PVY-NTN infection; PVYN-Wi infection specifically impacted cutin, suberine and wax biosynthesis, phenylalanine metabolism, phenylalanine, tyrosine and tryptophan biosynthesis, and monoterpenoid biosynthesis.

**Discussion:**

Results suggest that PVYN-Wi-induced necrosis may be mechanistically distinguishable from that of PVY-NTN. Our study provides a basis for understanding the mechanism underlying the development of PVY-induced tuber necrosis in potato.

## Introduction

Viral phytopathogens attack a wide range of crops worldwide (Song et al., 2010), resulting in economic losses of nearly US $60 billion annually. In potatoes (*Solanum tuberosum* L.), several viral pathogens have global significance (Scholthof et al., 2011), of which potato virus Y (PVY) causes the greatest crop losses (Lacomme et al., 2017). Taxonomically, PVY is a member of the genus *Potyvirus*, which comprises nearly 200 species that are transmitted by aphids (Harrington et al., 1986). Since non-persistent transmission occurs during brief feeding probes, even highly efficient aphicides fail to suppress PVY spread by non-colonizing aphids (Perring et al., 1999).

The PVY virion contains a 9.7-kb single-stranded, positive-sense RNA genome (Dougherty and Carrington, 1988) with two opening reading frames (ORFs) that encode at least 11 proteins. The larger ORF is translated into a single polyprotein composed of 10 functional proteins. During maturation, this polyprotein is cleaved by the proteinase activities of three of the virally coded proteases namely, P1 (the first protein at the N-terminus of the polyprotein), HC-Pro (helper component‐proteinase), and NIa-Pro (nuclear inclusion protein a), leading to the release of 10 independent, fully functional proteins (Revers and García, 2015; Lacomme et al., 2017).

The other and shorter ORF called PIPO (Pretty Interesting Potyviridae ORF) occurs within the P3 cistron but in two different reading frames relative to the polyprotein ORF, producing two distinct proteins called P3N-PIPO and P3N-ALT. In the case of P3N-PIPO, PIPO is translated *via* a −1 slippage of the viral RNA-dependent RNA polymerase (RdRp) in the P3 cistron. The resulting fusion protein (P3N-PIPO) consists of the N-terminal half of P3 (P3N) and the product (PIPO) of the −1 pipo ORF (Chung et al., 2008; Olspert et al., 2015). However, P3N-ALT is produced *via* a +1 slippage of the RdRp and is thus considered to be a C-terminal truncated form of P3 (Karasev and Gray, 2013; Hagiwara-Komoda et al., 2016). Considerable information has been published on the functional aspects of these potyviral proteins in the infection cycle of PVY and other potyviruses. Perhaps unsurprisingly, most of them are known to play multifunctional roles, which compensates for the low coding capacity of the viral genome (Martínez and Daròs, 2014).

Like most RNA viruses, PVY displays a remarkable genetic diversity; at least nine strains have been described to date (Piche et al., 2004; Gray et al., 2010; Karasev and Gray, 2013). As a result, significant shifts in strain co-circulation in potato crops have been observed, especially in Europe and North America, where newer strains named PVY^NTN^ and PVY^N-Wi^ have become more widespread (Shrestha et al., 2014). The most notable differences between PVY^NTN^ and PVY^N-Wi^ relate to the number of recombination junctions in each genome (Piche et al., 2004; Gray et al., 2010; Karasev and Gray, 2013; Revers and García, 2015) and serological reactivity (Karasev et al., 2010), as well as long-distance transport in the host plant (Dupuis et al., 2019). Within the PVY^NTN^ strain group, some recombinants contain three recombination junctions located in the HC-Pro/P3, VPg, and CP regions of the genome (Karasev and Gray, 2013), while other isolates contain an additional recombination junction in the P1 encoding region (Gao et al., 2015). PVY^N-Wi^ isolates can be divided into two groups based on the recombination patterns. One group contains two recombination junctions (in the P1 and HC-Pro/P3 encoding regions), and the second group, designated as PVY^N:O^ in North America (Gray et al., 2010), has only one (in the HC-Pro/P3 encoding region) (Piche et al., 2004; Karasev and Gray, 2013).

Of greater concern, however, is the virulence of these newly identified PVY strains. Unlike their progenitor strains, PVY^N^ and PVY°, both recombinants cause significant tuber damage to susceptible cultivars. Specifically, PVY^NTN^ infections are associated with potato tuber necrotic ringspot disease (PTNRD), while PVYN-Wi induces tuber cracking (Karasev and Gray, 2013; Benedict et al., 2015). These necrotic phenotypes reduce crop yield and quality and the marketability of the affected tubers (Beczner et al., 1984; Romancer et al., 1994). The metabolomic profiles of two potato cultivars that differ in their response to PVY° and the necrosis-causing strains PVY^NTN^ and PVYN-Wi were reported recently (Manasseh et al., 2023). Moyo et al. (2017) reported the small RNA profiles in response to three biologically distinct PVY strains that included two necrosis-causing strains, PVY^N^ and PVY^NTN^. However, the molecular basis of this necrosis remains poorly understood.

To gain better insights into PVY–host interactions at the molecular level, mRNA-Seq was used to investigate the transcriptional and functional responses associated with compatibility between a PVY-susceptible cv. Russet Burbank and two necrotic strains and one of the progenitor strains of PVY. Findings provided some important insights into strain-specific and generic responses of Russet Burbank to PVY infection. A model for the mechanism of tuber necrosis induced by PVY is proposed.

## Results

### Throughput and quality of the transcriptomes generated by mRNA-Seq

Several benchmarks were used to assess the throughput and quality of sequencing reads including the total raw read count for each sample (library), the proportion of clean reads in the raw reads, their base-wise accuracy or sequencing error rate, and GC content. These metrics are summarized in **Table 1**.

**Table 1:** Average quality metrics for sequencing reads obtained for the inoculation treatments.

Treatment	Quality metric						
	Total reads (million)	Clean reads (million)	Clean bases	Error rate (%)	Q20	Q30	GC (%)
PVY°	44.78	43.92	6.6G	0.03	97.59	92.90	43.15
PVY^NTN^	46.19	55.56	8.3G	0.03	97.46	92.65	42.77
PVY^N-Wi^	56.45	45.42	6.8G	0.03	97.48	92.69	43.15
Mock	51.38	50.62	7.6G	0.03	97.27	92.22	43.03

Values are averages for three replications of each treatment.

Clean reads, number of reads after filtering; Clean bases, number of clean reads multiplied by the read length and then converted to G as the unit; Error rate, average sequencing error rate, calculated by the formula: Qphred = −10log10 (E); Q20 and Q30, the percentage of bases for which the Phred value was larger than 20 and 30, respectively; GC content, the sum of the number of bases G and C, calculated as a percentage of the total base number.

In terms of overall throughput, 596.43 million raw reads were generated from the four libraries. After filtering, 586.56 million reads were retained as clean reads, which averaged over 40 million per replicate or sample library (**Table 1**). The mean single base error rate was lower than 1%, and GC content per read averaged 42%–43%. The Q20 scores (percentage of bases whose base call accuracy exceeds 99%) averaged 97%, while the Q30 scores (percentage of bases whose base call accuracy exceeds 99.9%) averaged 92%.

### Mapping and alignment metrics of RNA-Seq reads

**Figure 1:**
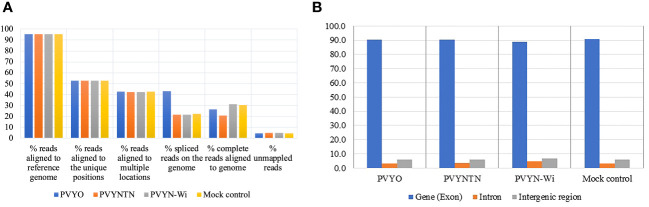
Mapping characteristics of reads from mRNA-Seq data from the three strains of potato virus Y (PVY): PVY°, PVY^NTN^, and PVY^N-Wi^. **(A)** Percent distributions of mapped reads considering overall alignment rates, unique and multi-aligned reads, spliced reads, and complete reads. **(B)** Distribution of mapped reads among exonic, intronic, and intergenic features of the reference genome.

In addition to the overall alignment rates, mapped reads were annotated in terms of the proportions of reads that aligned uniquely or to multiple loci, as well as the proportions of complete reads versus reads composed of spliced (or truncated) RNA. The results of the alignment are presented in **Figure 1A**.

Across all treatments, the alignments achieved read coverage rates of over 90%, with unmapped reads averaging 4.4%–6.1% of clean reads. Meanwhile, 52%–55% of reads mapped uniquely across treatments, whereas 40%–43% mapped to more than one locus. By comparison, the proportion of spliced reads averaged 20%–22% except for PVY°, which was significantly higher (43%). As a corollary, the fraction of complete reads for PVY° was lower than the 30% observed for PVY^N-Wi^ and mock treatment, although a similarly lower annotation of complete reads was also observed for PVY^NTN^.

The aggregate distribution of mapped reads over exonic, intronic, and intergenic features of the reference genome was another mapping parameter generated (Oshlack et al., 2010). The resulting distribution statistics are shown in **Figure 1B**. As shown, exons are the highest fraction of mapped reads (88%–91%), followed by intergenic regions (5.7%–6.6%), with 3.4%–4.6% of the reads falling within introns. No significant differences in reads mapping to these genome features were found among the inoculation treatments.

### Differential gene expression as measured by quantitative reverse transcription-PCR

**Figure 2:**
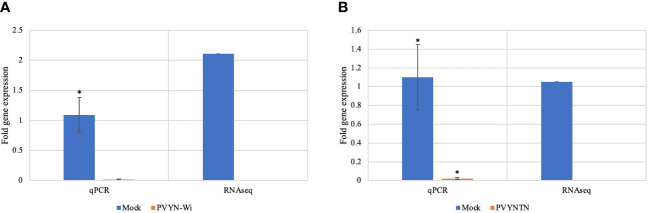
qRT-PCR validation of expression levels of putative DEGs identified by mRNA-Seq. **(A)** Downregulated expression of omega-hydroxypalmitate O-feruloyl transferase (OHFT) after PVY^N-Wi^ inoculation. **(B)** Downregulated expression of serine hydroxymethyl transferase (SHMT) after PVY^NTN^ inoculation. The independent samples t-test at p < 0.05 was used as threshold for statistical significance. The bar graphs show fold change observed between mock and PVYN-WI after inoculation. The qPCR data show an average of onefold downregulation in PVY infection tissue, while the actual RNA-Seq data show over twofold downregulation for gene OHFT; for gene SHMT, the onefold downregulation is noticeable in both qPCR and RNA-Seq data. DEGs, differentially expressed genes.

To validate the differential gene expression patterns related to PVY infection as determined by RNA-Seq, the expression levels of selected candidate differentially expressed genes (DEGs) were determined by quantitative reverse transcription-PCR (qRT-PCR). The expression trends obtained by qRT-PCR were consistent with those of mRNA-Seq, further supporting the accuracy and reliability of the sequencing data (**Figure 2**).

### Gene co-expression patterns induced by PVY strains

**Figure 3:**
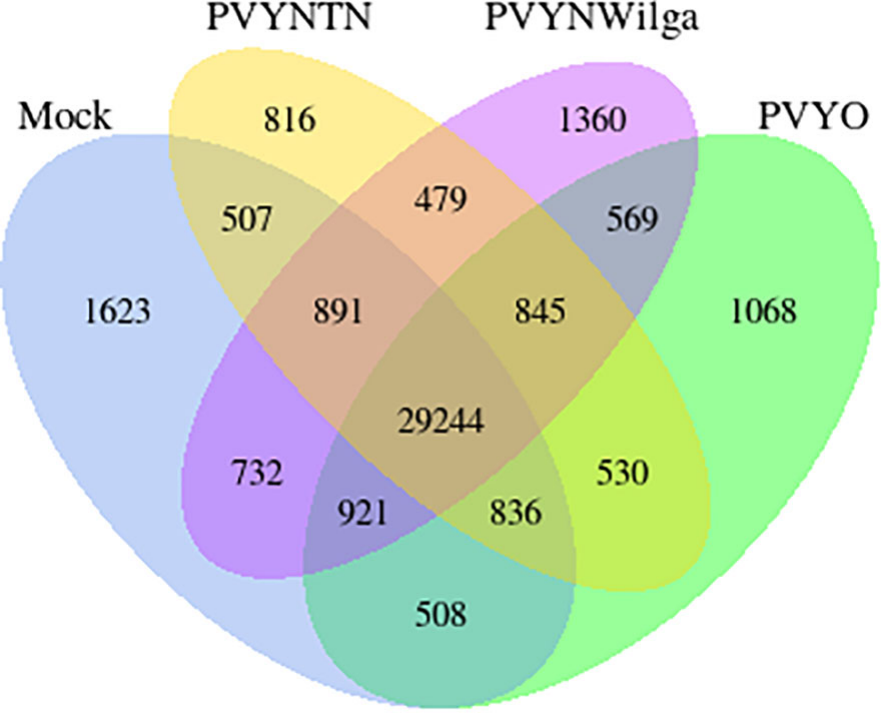
Venn diagram showing distribution of genes with similar and unique expression levels under mock, Potato virus Y (PVY)^NTN^, PVY^N-Wi^, and PVY° inoculation of Russet Burbank. The non-overlapping portions represent the number of genes that are uniquely expressed within each treatment group, with the overlapping regions showing the number of genes that are co-expressed under two or more treatment groups. An expression threshold of 0.3–1 FPKM (FPKM > 1) was used. For example, 1,068 genes were uniquely expressed in PVY° group, 569 genes were co-expressed in PVY° and PVY^N-Wilga^, and 29,244 genes were commonly expressed among mock, PVY^NTN^, PVY^N-Wilga^, and PVY°. FPKM, fragments per kilobase of transcript per million mapped reads.

Gene co-expression across the PVY treatments was assessed based on the per-gene read counts [fragments per kilobase of transcript per million mapped reads (FPKM)] obtained from the FeatureCounts program as input data. The resulting pattern is shown in **Figure 3**.

As **Figure 3** illustrates, 569, 530, and 479 virus-responsive genes were found to be co-expressed between PVY° and PVY^N-Wi^, PVY° and PVY^NTN^, and PVY^NTN^ and PVY^N-Wi^, respectively. All three PVY strains shared an overall total of 845 co-expressed genes. The number of uniquely expressed genes was the largest for the mock inoculation (1,623), followed by PVY^N-Wi^ (1,360), PVY° (1,068), and PVY^NTN^ infection (816). In the context of susceptibility, these overlapping and strain-specific gene sets may be factors in the differences in strain virulence displayed by PVY.

### Differential gene expression patterns induced by PVY inoculation

**Figure 4:**
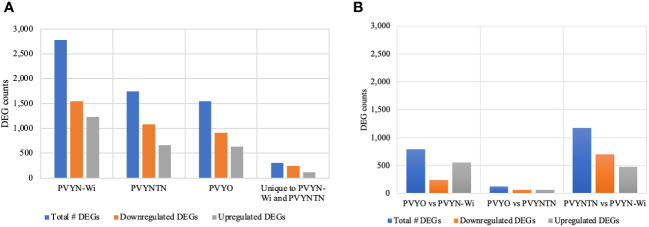
Significant DEGs induced (or repressed) following infection by the different potato virus Y (PVY) strains. **(A)** Differential gene expression in PVY-inoculated samples relative to mock-inoculated samples. **(B)** Differential gene expression between PVY-inoculated samples. FDR/padj ≤ 0.05 and |log2FoldChange| ≥ 1.0 were used for selection of DEGs. DEGs, differentially expressed genes; FDR, false discovery rate.

In addition to co-expression analysis on virus-responsive genes, differential analysis with the read count matrix was employed to identify genes whose expression levels were significantly altered by PVY infection. Gene expression levels were considered significant at false discovery rate (FDR) ≤0.05 and log2 ratio ≥1. **Figure 4** is a graphic representation of the DEG counts.

Total DEG counts varied among strains, being the highest under PVY^N-Wi^ treatment (2,778), followed by PVY^NTN^ (1,744), and the lowest under PVY° infection (1,549) (**Figure 4A**). Across the three PVY-mock comparisons, the number of DEGs under downregulation was greater than that under upregulation. Under PVY° infection, for example, 913 DEGs were downregulated and 636 were upregulated. In the case of PVY^NTN^ infection, 1,083 DEGs were downregulated and 661 were upregulated. Of the 2,778 DEGs induced by PVY^N-Wi^, 1,546 were downregulated and 1,232 were upregulated. On aggregate, the three PVY strains yielded 6,071 DEGs. Among these, 306 overlapped between PVY^N-Wi^ and PVY^NTN^ only, with 242 being downregulated and 112 upregulated.

Among the three PVY strains, the total DEG count (**Figure 4B**) was the highest between PVY^NTN^ and PVY^N-Wi^ (1,175), followed by PVY° and PVY^N-Wi^ (794), and PVY° versus PVY^NTN^ (128). In terms of the direction of differential expression, the number of DEGs downregulated was greater only between PVY^NTN^ and PVY^N-Wi^. Between PVY° and PVY^N-Wi^, fewer DEGs were downregulated. In the case of PVY° and PVY^NTN^, the number of up- and downregulated DEGs was comparable.

### Significant GO terms associated with PVY infection

**Figure 5:**
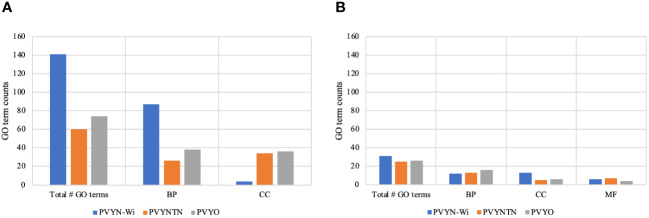
GO term structure associated with potato virus Y (PVY) inoculations. **(A)** GO term enrichment for downregulated DEGs. **(B)** GO term enrichment for upregulated DEGs. BP, CC, and MF refer to biological process, cellular component, and molecular function, respectively. GO, Gene Ontology; DEGs, differentially expressed genes.

To characterize the biological significance of the DEGs induced by PVY infection, enrichment or over-representation analysis of Gene Ontology (GO) terms was first performed on the DEG sets for each strain. A total of 357 GO terms were annotated across the three PVY treatments, and their distribution is given in **Figure 5**.

As shown in **Figure 5**, a total of 100 GO terms were enriched for the DEGs related to PVY°. Among these, 74 GO terms were enriched for the downregulated DEGs and comprised 38 biological processes and 36 molecular functions (**Figure 5A**). The remaining 26 GO terms were enriched for the upregulated DEGs and included 16 biological processes, six cellular components, and four molecular functions (**Figure 5B**).

For the DEGs associated with PVY^NTN^ infection, 85 GO terms were annotated. Of these, 60 GO terms were enriched for the downregulated DEGs and included 26 biological processes and 34 molecular functions (**Figure 5A**). Further, 25 GO terms were enriched for the upregulated DEGs and included 13 biological processes, five cellular components, and seven molecular functions (**Figure 5B**).

Among the three PVY strains, 172 GO terms were enriched in response to PVY^N-Wi^ inoculation. The majority of these (141 GO terms) were enriched for the downregulated DEGs and included 87 biological processes, four cellular components, and 50 molecular functions (**Figure 5A**). By contrast, 31 GO terms were enriched for the upregulated DEGs and included 12 biological processes, 13 cellular components, and six molecular functions (**Figure 5B**).

Whereas the overall GO term representation differed among the three PVY strains, the analysis also identified a subset of 36 common GO terms (**Table 2**). Among them, 31 GO terms were enriched for downregulated DEGs, with 15 being biological processes and the other 16 molecular functions.

**Table 2:** Significant GO terms that overlapped among all three strains of potato virus Y (PVY): PVY°, PVY^NTN^, and PVY^N-Wi^.

Category	GOID	Description	GeneRatio	padj	Count
GO terms enriched for downregulated DEGs					
BP	GO:0008610	Lipid biosynthetic process	21/362	0.0002	21
BP	GO:0006694	Steroid biosynthetic process	8/362	0.0002	8
BP	GO:0008202	Steroid metabolic process	8/362	0.0002	8
BP	GO:0016125	Sterol metabolic process	4/362	0.0015	4
BP	GO:0016126	Sterol biosynthetic process	4/362	0.0015	4
BP	GO:0008299	Isoprenoid biosynthetic process	7/362	0.0024	7
BP	GO:0006720	Isoprenoid metabolic process	7/362	0.0026	7
BP	GO:0006721	Terpenoid metabolic process	4/362	0.0124	4
BP	GO:0016114	Terpenoid biosynthetic process	4/362	0.0124	4
BP	GO:0016052	Carbohydrate catabolic process	9/362	0.0227	9
BP	GO:0005976	Polysaccharide metabolic process	14/362	0.0012	14
BP	GO:1901617	Organic hydroxy compound biosynthetic process	9/362	0.0000	9
BP	GO:1901615	Organic hydroxy compound metabolic process	9/362	0.0004	9
BP	GO:0051186	Cofactor metabolic process	22/362	0.0026	22
BP	GO:0006732	Coenzyme metabolic process	13/362	0.0130	13
MF	GO:0016702	Oxidoreductase activity, acting on single donors with incorporation of molecular oxygen, incorporation of two atoms of oxygen	8/481	0.0027	8
MF	GO:0016701	Oxidoreductase activity, acting on single donors with incorporation of molecular oxygen	8/481	0.0068	8
MF	GO:0016628	Oxidoreductase activity, acting on the CH-CH group of donors, NAD or NADP as acceptor	4/481	0.0032	4
MF	GO:0051213	Dioxygenase activity	8/481	0.0027	8
MF	GO:0016229	Steroid dehydrogenase activity	4/481	0.0047	4
MF	GO:0003854	3-Beta-hydroxy-delta5-steroid dehydrogenase activity	4/481	0.0047	4
MF	GO:0033764	Steroid dehydrogenase activity, acting on the CH-OH group of donors, NAD or NADP as acceptor	4/481	0.0047	4
MF	GO:0046527	Glucosyltransferase activity	13/481	0.0007	13
MF	GO:0016758	Transferase activity, transferring hexosyl groups	16/481	0.0017	16
MF	GO:0016769	Transferase activity, transferring nitrogenous groups	4/481	0.0040	4
MF	GO:0008483	Transaminase activity	4/481	0.0040	4
MF	GO:0008171	*O*-Methyltransferase activity	8/481	0.0040	8
MF	GO:0003950	NAD+ ADP-ribosyl transferase activity	3/481	0.0462	3
MF	GO:0042910	Xenobiotic transmembrane transporter activity	10/481	0.0046	10
MF	GO:2001070	Starch binding	3/481	0.0069	3
MF	GO:0016872	Intramolecular lyase activity	6/481	0.0004	6
GO terms enriched for upregulated DEGs					
CC	GO:0000786	Nucleosome	8/68	0.0000	8
CC	GO:0044815	DNA packaging complex	8/68	0.0000	8
CC	GO:0032993	Protein–DNA complex	8/68	0.0000	8
CC	GO:0000785	Chromatin	8/68	0.0000	8
CC	GO:0044427	Chromosomal part	8/68	0.0001	8

GeneRatio, padj, and count values are for PVY° inoculation.

GeneRatio, differential gene count in this GO term versus total differential gene count; padj, adjusted p-value; Count, differential gene count; GO, Gene Ontology; DEGs, differentially expressed genes; BP, biological process; MF, molecular function; CC, cellular component.

Of the 15 biological processes, five were related to lipid metabolism, including the lipid biosynthetic process (GO:0008610), steroid biosynthetic process (GO:0006694), steroid metabolic process (GO:0008202), sterol metabolic process (GO:0016125), and sterol biosynthetic process (GO:0016126). Also detected were isoprenoid biosynthetic process (GO:0008299), isoprenoid metabolic process (GO:0006720), terpenoid metabolic process (GO:0006721), and terpenoid biosynthetic process (GO:0016114), all of which participate in terpenoid metabolism. Carbohydrate catabolic process (GO:0016052) and polysaccharide metabolic process (GO:0005976) were shared GO terms related to carbohydrate metabolism. Likewise, organic hydroxy compound biosynthetic process (GO:1901617) and organic hydroxy compound metabolic process (GO:1901615) were the only GO terms related to the metabolism of organic hydroxy compounds. The other shared biological processes were cofactor metabolic process (GO:0051186) and coenzyme metabolic process (GO:0006732), which are necessary for the proper functioning of enzymes.

Of the 16 shared molecular functions, four had transferase activity annotation, including glucosyltransferase activity (GO:0046527) transferase activity, transferring hexosyl groups (GO:0016758) transferase activity, transferring nitrogenous groups (GO:0016769), *O*-methyltransferase activity (GO:0008171), and NAD+ ADP-ribosyl transferase activity (GO:0003950). A further four were annotated with steroid dehydrogenase activity, including steroid dehydrogenase activity (GO:0016229), 3-beta-hydroxy-delta5-steroid dehydrogenase activity (GO:0003854), and steroid dehydrogenase activity, acting on the CH-OH group of donors, NAD or NADP as acceptor (GO:0033764). Three molecular functions had oxidoreductase activity, including oxidoreductase activity, acting on single donors with incorporation of molecular oxygen, incorporation of two atoms of oxygen (GO:0016702); oxidoreductase activity, acting on the CH-CH group of donors, NAD or NADP as acceptor (GO:0016628); and oxidoreductase activity, acting on single donors with incorporation of molecular oxygen (GO:0016701). The other overlapping molecular functions were intramolecular lyase activity (GO:0016872), dioxygenase activity (GO:0051213), transaminase activity (GO:0008483), xenobiotic transmembrane transporter activity (GO:0042910), and starch binding (GO:2001070).

As shown in **Table 2**, the five GO terms that were enriched for upregulated DEGs were all cellular components that included nucleosome (GO:0000786), DNA packaging complex (GO:0044815), protein–DNA complex (GO:0032993), chromatin (GO:0000785), and chromosomal part (GO:0044427).

In contrast to these GO terms that enriched in all three treatments, there were 10 GO terms that overlapped uniquely between PVY^N-Wi^ and PVY^NTN^ only (**Table 3**). Of these, six were molecular functions that all enriched for downregulated DEGs and included 1-deoxy-d-xylulose-5-phosphate synthase activity (GO:0008661); transferase activity, transferring aldehyde or ketonic groups (GO:0016744); lyase activity (GO:0016829); pyridoxal phosphate binding (GO:0030170); vitamin B6 binding (GO:0070279); and vitamin binding (GO:0019842).

**Table 3:** Significant GO terms that overlapped uniquely between the two tuber necrotic strains of potato virus Y (PVY): PVY^NTN^ and PVY^N-Wi^.

GO terms enriched for downregulated DEGs					
Category	GOID	Description	GeneRatio	padj	Count
MF	GO:0008661	1-Deoxy-d-xylulose-5-phosphate synthase activity	4/843	0.0023	4
MF	GO:0030170	Pyridoxal phosphate binding	15/843	0.0154	15
MF	GO:0070279	Vitamin B6 binding	15/843	0.0154	15
MF	GO:0019842	Vitamin binding	15/843	0.0363	15
MF	GO:0016829	Lyase activity	26/843	0.0035	26
MF	GO:0016744	Transferase activity, transferring aldehyde or ketonic groups	4/843	0.0023	4
GO terms enriched for upregulated DEGs					
BP	GO:0005992	Trehalose biosynthetic process	5/307	0.0358	5
BP	GO:0005991	Trehalose metabolic process	5/307	0.0424	5
BP	GO:0046351	Disaccharide biosynthetic process	5/307	0.0488	5
MF	GO:0043565	Sequence-specific DNA binding	23/529	0.0005	23

GeneRatio, differential gene count in this GO term versus total differential gene count; padj, adjusted p-value; Count, differential gene count; GO, Gene Ontology; DEGs, differentially expressed genes; MF, molecular function; BP, biological process.

The other four GO terms were enriched for upregulated DEGs and comprised three biological processes and one molecular function (**Table 2**). Two of the three biological processes, trehalose biosynthetic process (GO:0005992) and trehalose metabolic process (GO:0005991), are related to trehalose metabolism. The remaining biological process, disaccharide biosynthetic process (GO:0046351), participates in the formation of disaccharides. The only molecular function, sequence-specific DNA binding (GO:0043565), is related to the regulation of gene expression.

**Figure 6:**
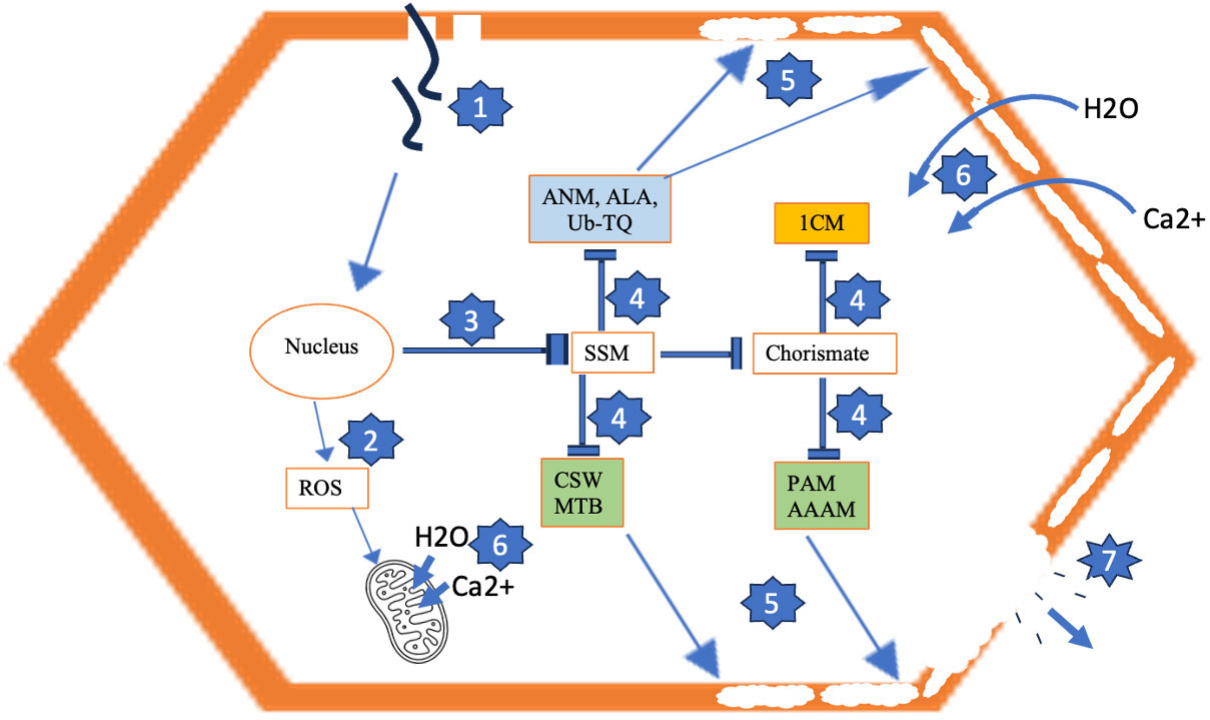
Model for development of tuber necrosis induced by PVY. 1 = Virus infection. 2 = Virus-induced ROS accumulation, resulting in mitochondrial membrane permeabilization. 3 = Repression of starch and sucrose metabolism (SSM) and cascading pathways that produce inputs for cell wall biosynthesis (4), resulting in permeabilized cell wall due to improper deposition of cell wall components (5). 6 = Ca^2+^ and H_2_O influx across permeabilized cell wall and mitochondrial membrane, leading to excessive turgor pressure and cell rupture and loss of cell contents (7). Blue-shaded pathways are generic to PVY^NTN^ and PVY^N-Wi^. Brown-shaded pathway is uniquely downregulated by PVY^NTN^. Green-shaded pathways are uniquely repressed by PVY^N-Wi^. ANM, amino sugar and nucleotide sugar metabolism; ALA, α-linolenic acid metabolism; Ub-TQ, ubiquinone and other terpenoid-quinone biosynthesis; CSW, cutin, suberine and wax biosynthesis; MTB, monoterpenoid biosynthesis; 1CM, one-carbon metabolism; PAM, phenylalanine metabolism; AAAM, aromatic amino acid metabolism; ROS, reactive oxygen species.

Diverse sets of overrepresented GO terms were also observed among the PVY strains and are presented in **Figure 6**. For the DEGs between PVY° and PVY^NTN^, the downregulated DEGs yielded no GO term. In the case of upregulated genes, the analysis yielded one significant biological process (copper ion binding, GO:0005507). For the DEGs between PVY° and PVY^N-Wi^, no GO term was annotated for the downregulated DEGs. However, 41 GO terms were enriched for the upregulated DEGs, of which 29 terms were biological processes and 12 were molecular functions. For the DEGs between PVY^NTN^ and PVY^N-Wi^, the downregulated DEGs were involved in 36 GO terms, of which 20 terms were biological processes and 16 were molecular functions. However, two significant GO terms were enriched for the upregulated genes, both of which were molecular functions (GO:0016799, hydrolase activity, hydrolyzing *N*-glycosyl compounds; GO:0019104, DNA *N*-glycosylase activity).

### Significant KEGG pathways modulating PVY compatibility with potato

None of the three PVY treatments resulted in the Kyoto Encyclopedia of Genes and Genomes (KEGG) pathway enrichment for upregulated DEGs.

**Figure 7:**
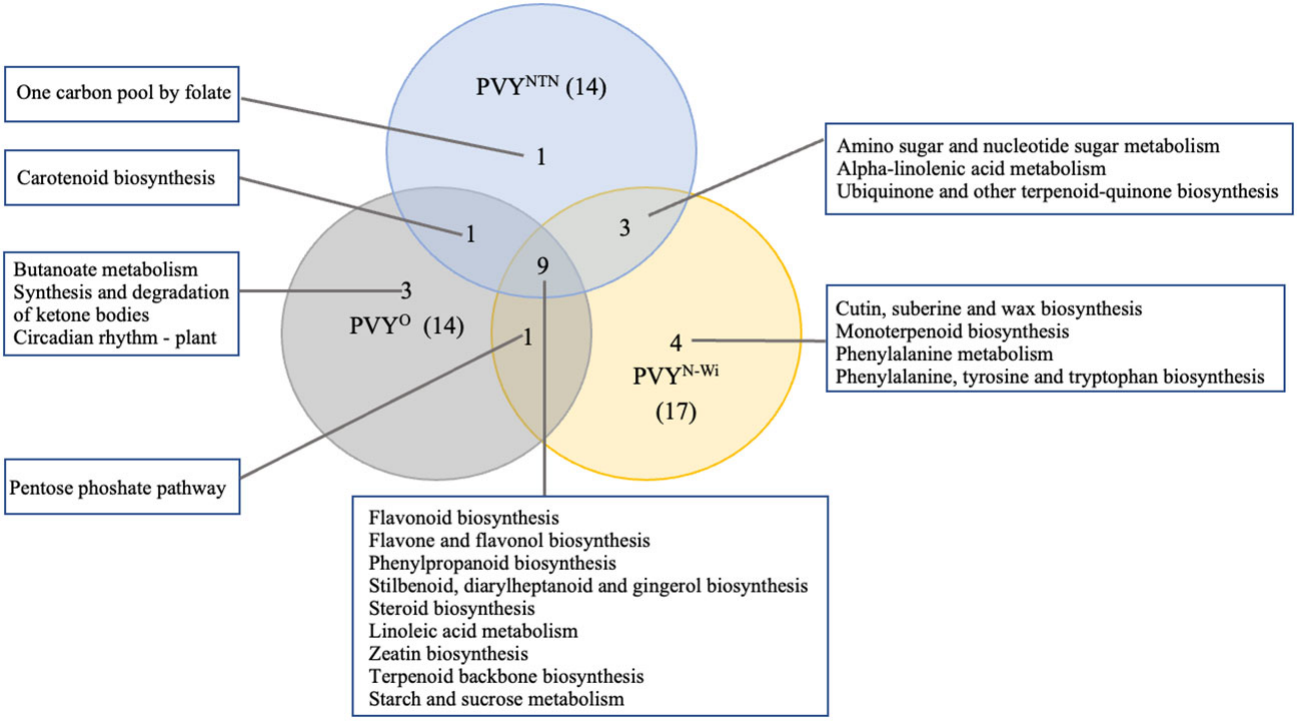
KEGG pathway overlaps among three strains of potato virus Y (potato virus Y): PVY°, PVY^NTN^, and PVY^N-Wi^. KEGG, Kyoto Encyclopedia of Genes and Genomes.

By contrast, annotation of the downregulated DEGs yielded a combined total of 22 different significant KEGG pathways. A comparison of these pathways among the PVY treatments is shown in **Figure 7**.

As shown in **Figure 7**, 14, 14, and 17 virus-responsive pathways were annotated for PVY°, PVY^NTN^, and PVY^N-Wi^, respectively. Among them, nine pathways were shared by all three PVY strains. This suggests that these pathways are active under PVY-related stress. Of these, four were related to biosynthesis of other secondary metabolites, including flavonoid biosynthesis (KEGGID: sot00941), flavone and flavanol biosynthesis (KEGGID: sot00944), phenylpropanoid biosynthesis (KEGGID: sot00940), and stilbenoid, diarylheptanoid and gingerol biosynthesis (KEGGID: sot00945). Steroid biosynthesis (KEGGID: sot00100) and linoleic acid metabolism (KEGGID: sot00591) were two pathways related to lipid metabolism. The remaining pathways across the three strains that overlapped were zeatin biosynthesis (KEGGID: sot00908), terpenoid backbone biosynthesis (KEGGID: sot00900), and starch and sucrose metabolism (KEGGID: sot00500). Only one pathway, carotenoid biosynthesis (KEGGID: sot00906), was shared between PVY^NTN^ and PVY°. Likewise, PVY° and PVY^N-Wi^ shared only the pentose phosphate pathway (KEGGID: sot00030). By contrast, PVY^N-Wi^ and PVY^NTN^ shared three pathways: amino sugar and nucleotide sugar metabolism (KEGGID: sot00520), alpha-linolenic acid metabolism (KEGGID: sot00592), and ubiquinone and other terpenoid-quinone biosynthesis (KEGGID: sot00130).

In addition to these overlaps, seven pathways were enriched in a strain-specific manner. Three of these were enriched for PVY°: butanoate metabolism (KEGGID: sot00650) related to carbohydrate metabolism, synthesis and degradation of ketone bodies (KEGGID: sot00072) linked to lipid metabolism, and circadian rhythm—plant (KEGGID: sot04712) linked to environmental adaptation. One carbon pool by folate (KEGGID: sot00670) was enriched exclusively for PVY^NTN^, while PVY^N-Wi^ impacted only cutin, suberine and wax biosynthesis (KEGGID: sot00073), monoterpenoid biosynthesis (KEGGID: sot00902), phenylalanine metabolism (KEGGID: sot00360), and phenylalanine, tyrosine and tryptophan biosynthesis (KEGGID: sot00400).

In addition to the pathways enriched between PVY and mock control, we evaluated pairwise enrichment between the necrotic strains and PVY°. No significant pathways were enriched for DEGs between PVY° and PVY^NTN^. By contrast, five KEGG pathways for the DEGs detected between PVY° and PVY^N-Wi^ were statistically significant. Fructose and mannose metabolism (KEGGID: sot00051) and pentose phosphate pathway (KEGGID: sot00030) were enriched for the downregulated DEGs. Conversely, the plant MAPK signaling pathway (KEGGID: sot04016), amino sugar and nucleotide sugar metabolism (KEGGID: sot00520), and phenylpropanoid biosynthesis (KEGGID: sot00940) were three pathways enriched for the upregulated DEGs.

## Discussion

### Overview of the study approach and transcriptome assemblies

As a rule, plant–pathogen interactions are associated with quantifiable changes in the host transcriptome, proteome, metabolome, and epigenome (Garcia-Seco et al., 2017). Thus, omics studies have been widely adopted in researching disease pathogenesis, as they can reveal not only the underlying mechanisms of plant resistance but also the strategies used by the pathogen leading to host susceptibility and the subsequent establishment of infection.

In the present study, the response of PVY-susceptible cv. Russet Burbank to infection by strains PVY^NTN^, PVY^N-Wi^, and PVY° was characterized by mRNA-Seq. Unlike their common progenitor PVY°, both PVY^NTN^ and PVY^N-Wi^ are major triggers of tuber necrosis in susceptible potato cultivars. However, the molecular mechanisms inducing necrosis remain poorly understood.

Bioinformatics analysis of the mRNA-Seq data was carried out using the recently published autotetraploid genomes (Hoopes et al., 2022) to explore key aspects of the host–virus interaction, including the differential gene expression outcomes of PVY infection, and their functional significance in inducing host susceptibility and necrosis in the potato host. The throughput and quality of the sequencing reads obtained were within the range considered suitable for high-quality mRNA/whole transcriptome profiling. For example, over 90% of the total clean reads are mapped to the reference genome. The proportion of clean reads obtained, which averaged over 40 million per sample, was also within the 40–60 million range for paired-end reads required for higher-accuracy sequencing projects, including studies of alternate splicing (Conesa et al., 2016).

Whereas over 90% of the reads aligned to the reference genome, unmapped reads averaging 4.4%–6.1% of the clean reads from the four libraries were also detected across all treatments. The significance of these unmapped reads warrants further exploration, as their elucidation could reveal new and meaningful biological information (Laine et al., 2019), including sequences related to the studied PVY strains and genes missing in the reference genome (Isakov et al., 2011; Samuels et al., 2013). For this, *de novo* assemblies could be generated from the unmapped reads, followed by BLAST alignment of the generated sequence contigs to the National Center for Biotechnology Information (NCBI) non-redundant nucleotide database, which can reveal known matching sequences (Laine et al., 2019).

Although most of the clean reads mapped uniquely to the two genomes, a significant fraction (40%–43%) also mapped to multiple positions. The relatively high multi-mapping rate can be attributed to the autotetraploid nature of the reference genome used (Hoopes et al., 2022), as well as the high rates of gene-level copy number variation generally encountered in tuber-bearing *Solanum* plants (Hardigan et al., 2017).

A significant fraction (20%–22%) of mapped reads were annotated as spliced chimeric reads. This is not surprising, as eukaryotic genomes generally contain large numbers of introns interspersed among the coding sequences (exons) of their genes. In plants, nearly 60% of such genes are subject to alternative splicing (Filichkin et al., 2010; Mandadi and Scholthof, 2015), where some intronic sequences present in pre-mRNA can be retained in, or removed, from mature mRNA. Consequently, many mRNA variants are produced from one gene (Ben-Dov et al., 2008), which makes alternative splicing a major source of cellular transcriptome and proteome diversity (Pan et al., 2008; Nilsen and Graveley, 2010). In the present study, the proportion of spliced reads obtained from PVY°-treated plants was noticeably higher than observed for PVY^NTN^ and PVY^N-Wi^. Further investigation is required to determine whether these splicing patterns contribute to the added virulence of PVY^NTN^ and PVY^N-Wi^ over the PVY° strain.

Other mapping parameters that were generated included the aggregate distribution of mapped reads over exonic, intronic, and intergenic features of the reference genome (Oshlack et al., 2010); this is essential to understanding the gene expression behavior. Further, it indicates the quality of a genome assembly. Exon-mapped reads were the most abundant type of reads, indicating that both reference genomes are well-annotated (Parekh et al., 2018).

The goal of this study was to determine the likely transcriptomic and functional features of PVY susceptibility and tuber necrosis in potatoes. Bioinformatics analyses were carried out to systematically analyze the patterns of gene co-expression, differential gene expression, gene ontology, and KEGG pathway enrichment, from which we infer the following conclusions.

### Co-expression and DEG profiles induced by PVY

Through co-expression analysis, 845 co-expressed genes induced by the three PVY strains were revealed in this study. As such, these genes may be delineated as a common gene expression signature of PVY infection in this cultivar (Goyer et al., 2015). The results of differential gene expression analysis on the quantification results indicate that the DEG set elicited by PVY^N-Wi^ inoculation was significantly larger (2,778) than the DEG counts under PVY^NTN^ (1,744) and PVY° (1,549). On this basis, PVY^N-Wi^ can be seen as the most aggressive (or virulent) of the three strains. Interestingly, downregulated genes dominated the DEG counts for all three PVY strains. Thus, downregulated gene expression may largely sustain PVY compatibility.

A common feature of such transcriptional modifications is the reported suppression of host defense responses, which are essential for virus replication and symptom development (Whitham et al., 2006). A previous study of the potato–PVY pathosystem (Ross et al., 2022) revealed widespread downregulation during the early response in Russet Burbank after PVY^N-Wi^ inoculation. Key features of this reported downregulation were genes involved in plant immunity, including plant immune signaling and viral suppression of the antiviral RNAi pathway (Amari et al., 2012).

Among the potyviral proteins, the helper component proteinase (HCPro) is a well-characterized suppressor of antiviral RNAi (Ivanov et al., 2016). Thus, HCPro-mediated suppression of RNAi may be an important molecular explanation for the downregulated gene expression induced by PVY infection of this cultivar. Additionally, viruses can also modify phytohormone levels to attenuate defense or development signaling and change the cellular conditions to favor their replication and spread (Mishra et al., 2020). The differential expression analysis also uncovered 306 genes that achieved DEG significance under both PVY^N-Wi^ and PVY^NTN^, which made them potential candidate genes for tuber necrosis in potatoes.

### General GO signatures of PVY susceptibility

When enrichment of GO terms of the DEG sets from each PVY-mock comparison were analyzed, the GO term profiles generally mirrored the strain behavior depicted by DEG analysis, where the GO term count elicited by PVY^N-Wi^ inoculation was also significantly greater (172) than the counts under inoculation with PVY^NTN^ (85) and PVY° (100). Analysis for similarity and differences between PVY treatments revealed 36 common GO terms across the three strains, of which 31 were enriched for downregulated DEGs and five were enriched for upregulated DEGs. Together, these overlapping GO terms could be considered essential for the susceptibility of potatoes to PVY.

Among the 31 shared GO terms enriched for downregulated DEGs, 15 were biological processes and the other 16 were molecular functions. Five of the 15 biological processes were related to lipid and steroid metabolism, including lipid biosynthetic process, steroid biosynthetic process, steroid metabolic process, sterol metabolic process, and sterol biosynthetic process. In general, plant steroids, including brassinosteroids (BRs) and their precursors (phytosterols), are known to play important roles in growth tolerance to different abiotic and biotic stresses (Divi & Krishna, 2009), and their role in inducing resistance to plant viruses has been reported previously (Yu et al., 2018). Thus, the downregulation of these processes may suggest that BR repression is a key factor in compatibility between potato and PVY.

Four biological processes, isoprenoid biosynthetic process, isoprenoid metabolic process, terpenoid metabolic process, and terpenoid biosynthetic process, participate in terpenoid metabolism. Terpenoids mediate plant responses to biotic and abiotic factors (Tholl, 2006; Nagegowda, 2010), including fine-tuning host–vector–virus interactions to facilitate vector transmission (Wamonje et al., 2020), and the cellular environment for viral replication (Wu et al., 2019). Thus, the observed repression of these terpenoid-related processes could modulate aphid transmission and pathology of PVY in a similar fashion.

### Proposed model for the necrotic phenotype induced by PVY

On the basis of the foliar transcriptome data, a model for the necrotic behavior of PVY^N-Wi^ and PVY^NTN^ is proposed. Based on plant morphology, at least two classes of programmed cell death can be distinguished, including vacuolar cell death and necrosis (Van Doorn et al., 2011). Vacuolar cell death is common during tissue and organ formation and elimination and occurs *via* the removal of cell contents by a combination of an autophagy-like process and the release of hydrolases from collapsed lytic vacuoles (Van Doorn et al., 2011).

Necrosis occurs mainly under abiotic stress and is distinguished from vacuolar cell death by mitochondrial dysfunction and early loss of protoplast integrity (Van Doorn et al., 2011; Minina et al., 2013). Mitochondrial dysfunction is characterized by the initial permeabilization of the mitochondrial membrane. This implies the formation of pores or channels across the membrane whose inner layer, at physiological homeostasis, acts as a negatively charged system. The resulting membrane polarity is essential for the proper functioning of the respiratory chain and the generation of ATP. Classically, permeabilization of the mitochondrial membrane triggers the rapid influx of Ca^2+^ ions and water into the mitochondrial matrix, resulting in loss of Ca^2+^ homeostasis, dissipation of membrane potential, and swelling and rupture of mitochondria (Scott and Logan, 2008). Oxidative stress is known to contribute to mitochondrial dysfunction, as reactive oxygen species (ROS)-mediated oxidation can cause damage to the mitochondrial DNA, membrane, and respiratory chain (Guo et al., 2013), leading to impaired oxygen consumption and a profound drop in intracellular ATP (Clarke, 1990; Leist and Jäättelä, 2001).

Permeabilization of the plasma membrane causes the early loss of protoplast integrity (Gao & Showalter, 1999; Van Doorn et al., 2011). Cell membrane permeabilization can be triggered by factors such as ROS bursts and associated oxidation of membrane lipids (Yadav et al., 2018), excessive osmotic (turgor) stress (Van Doorn et al., 2011), and pore-forming toxins secreted by pathogens (Brito et al., 2019). A link between cell membrane permeabilization and cell wall integrity is also suggested by the fact that an aberrant cell wall architecture could adversely alter cell wall/plasma membrane interactions and organization, including interruptions in material trafficking, cell wall signaling, and cell wall–plasma membrane cross-linking (Liu et al., 2015).

In the permeabilized state, the increased permeability triggers Ca^2+^ flux into the cytosol (Fink and Cookson, 2005), thereby creating an intracellular ionic imbalance that induces water inflow. Accumulation of Ca^2+^ and water to pathological levels leads to a cascade of excessive protoplast swelling and turgor stress (Trump and Berezesky, 1996), lysis of the plasma membrane (Kroemer et al., 2009) and its retraction from the cell wall, and cell death. Based on these characteristics, a proposed model for the development of tuber necrosis induced by PVY is shown in **Figure 6**.

Our data suggest that after entering the cell, all three PVY strains induce downregulation of a mix of pathways of primary and secondary metabolism, which appear to attenuate host defense responses and promote/favor virus survival, propagation, and transmission. As such, these pathways can be considered generic mechanisms of potato susceptibility to PVY infection.

Among them, the common pathway of primary metabolism is starch and sucrose metabolism. In both source and sink tissue cells, sucrose is a raw material for the production of energy and carbon skeletons required for the biosynthesis of amino acids, nucleotides, starch, structural carbohydrates, and defense-related metabolites (Stein and Granot, 2019). Thus, we believe that the observed viral suppression of starch and sucrose metabolism weakens the potato host by inducing starvation, impeding tuber starch accumulation, and decreasing carbon supply for induced defense responses (Engelsdorf et al., 2013).

The suppression of starch and sucrose metabolism also correlates with the eight downregulated pathways of secondary metabolism that, collectively, could limit the scale and effectiveness of any host defense response against PVY. We speculate on the key features of attenuated secondary metabolism that could support or be essential for compatible potato–PVY interaction and inferred these to include disorders in brassinosteroid and cytokinin homeostasis, RNAi suppression, vasiRNA-directed host gene silencing, PVY-induced ROS toxicity, attenuated JA-mediated defense signaling, impaired cell membrane integrity, and disrupted primary carbon flow for secondary metabolism.

Our data suggest that in addition to these generic pathways, PVY^N-Wi^ and PVY^NTN^ infections also degrade the activities of another eight pathways that are not affected by PVY° inoculation. Three of these pathways were shared between PVY^N-Wi^ and PVY^NTN^ and include amino sugar and nucleotide sugar metabolism, α-linolenic acid metabolism, and ubiquinone and other terpenoid-quinone biosynthesis. The other five pathways were enriched in a strain-specific manner, where 1C metabolism was impacted specifically by PVY^NTN^ infection, while cutin, suberine and wax biosynthesis, phenylalanine metabolism, phenylalanine, tyrosine, and tryptophan metabolism, and monoterpenoid biosynthesis pathway were impacted uniquely by PVY^N-Wi^ inoculation. A common consequence of these pathway changes is the generation and supply of substrates for cell wall construction. This supports the conclusion that the necrotic phenotypes induced by both strains of PVY are preceded by initial cell wall permeabilization, which satisfies the first criterion for plant necrosis. Since both strains also cause downregulation of starch and sucrose metabolism, interference with carbon flux for cell wall synthesis could be a key factor promoting its permeabilization.

As indicated earlier, we believe that this abnormal cell wall architecture can also abolish the cell membrane’s selective permeability, resulting in the cascade of excessive Ca^2+^ and water intake, cell swelling, organelle and protoplast deformation, and subsequent rupture and cell death, key events to tuber necrosis. At the same time, viral downregulation of defense-related pathways would attenuate the power of redox defense against PVY-induced ROS stress and induce the mitochondrial dysfunction necessary for cell and tuber necrosis.

Therefore, what distinguishes PVY^N-Wi^-induced necrosis mechanistically from that of PVY^NTN^? As indicated earlier, 1C metabolism was enriched specifically by PVY^NTN^ infection, while cutin, suberine and wax biosynthesis, phenylalanine metabolism, phenylalanine, tyrosine, and tryptophan metabolism, and monoterpenoid biosynthesis pathway were impacted uniquely by PVY^N-Wi^ inoculation. This deviation between PVY^N-Wi^ and PVY^NTN^ is intriguing, considering that the three aromatic amino acids (tryptophan, phenylalanine, and tyrosine) and folate, which mediates 1C metabolism (Kaiser & Leistner, 1990), are all synthesized from the same precursor chorismate (Knaggs, 2003). Thus, the primary difference in the necrotic behaviors of these two strains could be related to cutin, suberine and wax metabolism.

Thus, we speculate that the downregulation of this pathway causes suboptimal deposition of these cell wall components, resulting in weakened and permeabilized cell walls. Since the cell wall protects cells from lysing by excessive turgor pressure and osmotic disequilibrium resulting from the net influx of water due to salt imbalance, this cell wall configuration can induce excessive water uptake, cell swelling, and loss of cell wall recalcitrance to large turgor pressure, leading to rupture of cells on a tissue-scale. We speculate that this cell rupture mirrors growth cracking, as it also induces suberin deposition (Woolfson et al., 2022), resulting in the suberized tuber cracking symptom associated with PVY^N-Wi^ (Benedict et al., 2015).

In this model, the absence of manifestations of foliar necrosis would be related to the criteria for defining the source–sink relationship between aerial and underground organs. In the context of water supply, plants extract water through their roots and transport it to their leaves for photosynthesis. As such, roots could be considered sources of water, while leaves and other aerial tissues represent net sinks for water transported from the root system (White et al., 2016). In addition, tuber tissues with defective cell walls would be more vulnerable to cell swelling and the associated cellular cascade that leads to plant necrosis than leaf cells, as foliar tissues can release excess water from their tissues through transpiration.

## Conclusions

PVY remains an economically important viral pathogen of potatoes worldwide. Host resistance breeding is considered to be the most effective management strategy. In this study, we used mRNA to investigate how a PVY-susceptible potato cultivar responds to infection by three strains of PVY (PVY^NTN^, PVY^N-Wi^, and PVY°). Our analyses showed that PVY infections in potatoes lead to detectable changes in primary and secondary metabolism. The analysis revealed a core set of pathways that can be considered the primary drivers of PVY susceptibility in the susceptible cv. Russet Burbank. These include flavonoid biosynthesis, flavone and flavanol biosynthesis, phenylpropanoid biosynthesis, stilbenoid, diarylheptanoid and gingerol biosynthesis, steroid biosynthesis, linoleic acid metabolism, zeatin biosynthesis, terpenoid backbone biosynthesis, and starch and sucrose metabolism. Our study also identified 10 GO terms and eight other KEGG pathways that enriched uniquely for PVY^NTN^ and/or PVY^N-Wi^. These pathways could be targeted to develop genetic resistance to tuber necrosis in potatoes.

## Materials and methods

### Experimental design

The experimental design was described in our previous study (Manasseh et al., 2023). Briefly, the experimental units were single potted potato plants of PVY-susceptible cv. Russet Burbank grown under controlled greenhouse conditions (soil, 21° ± 2°C, with a photoperiod of 16 hours and relative humidity of 70%). PVY strain was the sole treatment factor. To delineate possible differences and commonalities between PVY strains, the strain factor was tested for differences in potato response to inoculation with PVY^NTN^, PVYN-Wi, or PVY°. Mock inoculation was performed with sodium phosphate buffer as a control.

To offset the effects of unanticipated micro-environment variations within the greenhouse, the potted plants were arranged in BugDorm-2400 Insect Rearing Tents (MegaView Science. Co. Ltd., Taichung, Taiwan) in a randomized complete block design, with one tent as a block. Four weeks after transplanting, two fully expanded compound leaves selected from the medium plant canopy were inoculated as previously described (Goyer et al., 2015). Each treatment (inoculation) had three replicates, the minimum recommended for RNA-Seq experiments (Conesa et al., 2016). At 7 days post‐inoculation (dpi), two leaflets, one from each inoculated compound leaf, were harvested for total RNA extraction and their subsequent use in Illumina sequencing (mRNA-Seq) and RT-qPCR analysis.

### Extraction of total RNA from leaf samples

Total RNA was extracted with a modified protocol that sequentially combined the use of TRIzol reagent (Life Technologies, Gaithersburg, MD, USA) with phenol-chloroform extraction. Following extraction with TRIzol, the air-dried RNA pellets were first dissolved in 500 μL of diethylpyrocarbonate (DEPC)-treated water by incubation at 55°C for 10 minutes. After incubation, 500 μL of a mixture containing buffer-saturated phenol (VWR Chemicals, Radnor, PA, USA), chloroform, and isoamyl alcohol (VWR International, USA) in the ratio of 25:24:1 (v/v/v) was added to the solution.

The resulting suspensions were then mixed by inverting each tube several times before centrifuging at 4°C and 12,000 *g* for 20 minutes. Following centrifugation, ~400 μL of each supernatant was transferred into a fresh 1.5-mL microcentrifuge tube. To each supernatant was added 80 μL of 1 M sodium acetate at pH 5.2 followed by 0.7 times the resulting cumulative volume (supernatant and 1 M sodium acetate) of cold 100% isopropyl alcohol. The tubes were inverted several times to mix the contents followed by incubation at −20°C for 30 minutes.

After incubation, the solutions were centrifuged at 4°C and 12,000 *g* for 20 minutes. The resulting supernatants were discarded, and 1 mL of 3 M sodium acetate (pH = 5.2) was added, followed by a 5-minute centrifugation at 4°C and 10,000 *g*. The supernatants were again discarded, and the pellets were washed three times by adding 1 mL of 75% ethanol, followed by gentle vortexing to resuspend the pellets, and then centrifugation at 4°C and 7,500 *g* for 5 minutes. Following the final wash, the ethanol was decanted, and the pellets were air-dried at room temperature for 10 minutes. As a final step, the dried pellets were dissolved in 30 μL of DEPC-treated water at room temperature for 10 minutes.

Prior to the preparation of the complementary DNA (cDNA) libraries, the RNA samples were DNase-treated (Ambion-Life Technologies, Waltham, MA, USA) to minimize the contribution of sequence reads derived from residual genomic DNA in each sample. RNA quality was then assessed using the Agilent Bioanalyzer 2100 system (Agilent Technologies, Santa Clara, CA, USA), which assigned an RNA integrity number (RIN) to each sample.

### Preparation of cDNA library for sequencing mRNA

The mRNA was purified from 1.5 μg of total RNA (RIN ≥ 7.0) using Oligo(dT) magnetic beads oligo(dT), according to the manufacturer’s instructions. The cDNA library was prepared from mRNA using the NEBNext^®^ Ultra™ RNA Library Prep Kit for Illumina^®^ (New England BioLabs, Inc., Ipswich, MA, USA).

Following purification, divalent cations were used to fragment the mRNA at high temperatures in NEBNext^®^ First Strand Synthesis Reaction Buffer (5X) (New England BioLabs, Inc., USA). After fragmentation, random hexamer primers and M-MuLV Reverse Transcriptase (RNase H-) were used to synthesize the first cDNA strands, with the single-stranded mRNA as a template.

Subsequently, second-strand cDNA was synthesized with dUTP-containing dNTPs, DNA polymerase I, and RNase H. Double-stranded cDNA products with overhangs converted into blunt-ended cDNA using T4 DNA polymerase and Klenow DNA polymerase. After the end-repair, the cDNA fragments were then monoadenylated on the 3′ ends, to which hairpin looped NEB Next Adaptors were ligated *via* T-overhangs at their 3′ ends. To select adaptor-ligated cDNA fragments of preferentially 370–420-bp length, the ligation products were purified with the AMPure XP system (Beckman Coulter, Beverly, MA, USA). After purification, the selected cDNA fragments were enriched by PCR performed with Phusion High-Fidelity DNA polymerase, universal PCR primers, and Index (X) primer containing an 8-bp index sequence that allows identification of each library. Finally, amplification products were purified (AMPure XP system) to create the final cDNA library, and library quality was assessed on the Agilent Bioanalyzer 2100 system, following the manufacturer’s instructions.

### Clustering and sequencing of the cDNA libraries

Index-coded cDNA libraries were clustered on a cBot Cluster Generation System using TruSeq PE Cluster Kit v3-cBot-HS (Illumina) according to the manufacturer’s instructions. After cluster generation, library preparations were sequenced on an Illumina NovaSeq platform, and 150-bp paired-end reads were generated. Library preparation, sequencing, and the subsequent bioinformatics analysis were performed at Novogene Corporation (Sacramento, CA, USA).

### Bioinformatics analyses

Analysis of the RNA-Seq data addressed the following questions: 1) How is the potato transcriptome modified by PVY infection? 2) What are the potential functional consequences of the transcriptome modifications associated with PVY infection? 3) To what extent could these transcriptomic and functional adjustments subserve the necrotic behavior of PVY^NTN^ and PVY^N-Wi^? 4) What unique pathways are associated with necrotic ringspot and tuber cracking?

To answer these questions, the analysis followed the basic steps for a reference-based RNA-Seq data analysis as described by Conesa et al. (2016). These included quality check and preprocessing of raw sequence reads, mapping of the processed reads to the potato reference genome, quantification of gene and transcript levels, assessment of the scope of PVY-induced differential gene expression, and functional annotation of the identified DEGs.

The analysis pipeline was run using the recently published tetraploid genome for Castle Russet as a reference. Castle Russet is resistant to PVY (Quick et al., 2020). The genome assembly and the corresponding annotation files were downloaded from Dryad Data (https://datadryad.org/). In this study, the genome assembly with phased pseudomolecules, unphased pseudomolecules, and unplaced scaffolds (CR_v2.0_asm.fasta.gz) was used. The corresponding set of working gene models (cr.working_models.pm.locus_assign.gff3.gz) was used for annotation.

### Quality check and preprocessing of raw sequence reads

After sequencing, FastQC (Ver. 0.11.9) was used to examine the sequencing quality of the paired-end raw reads from each sample based on sequence quality, GC content, the presence of adaptors, overrepresented k-mers and duplicated reads, PCR artifacts, or contaminations (Conesa et al., 2016). Following the evaluation of the FastQC results, in-house perl scripts were used to clean the raw reads by removing adapter sequences from the reads and low-quality reads with >10% uncertain (N) base calls, as well as those with more than 50% low-quality (Q-value ≤ 20) bases. All the downstream analysis steps were run on the clean reads.

### Mapping, assembly, and quantification of gene expression

The reference genome was indexed, and clean reads from each sample were mapped to the indexed reference genome using Hisat2 v2.0.5. For assembly of the mapped reads in each sample into full-length transcripts, the alignments of each file were passed to StringTie (v1.3.3b) (Pertea et al., 2016) with the corresponding reference annotation file, which guided the assembly process. Following the initial assembly, the assembled transcripts were merged by StringTie.

To quantify gene expression, FeatureCounts v1.5.0-p3 (Liao et al., 2014) counted the uniquely mapped read numbers mapped to each gene. For this, FPKM values, which consider the read count and length of each gene (Mortazavi et al., 2008), were computed as a measure of gene expression level (Trapnell et al., 2009). Co-expression analysis was then performed to evaluate the variability in the observed gene expression levels (FPKM) between treatments.

### Analysis of differential gene expression between treatments

To assess the scope of PVY-induced changes in host gene expression, the per-gene counts were used as input for differential gene expression analysis with the DESeq2 R package (1.20.0). In addition to determining the fold changes between PVY and mock treatments, the default Wald test in DESeq2 was used to test the significance of the observed differential expression. The resulting Wald test *p*-values were adjusted for multiple hypothesis testing using the Benjamini–Hochberg procedure to control the false discovery rate. Genes with an adjusted *p*-value ≤0.05, FDR < 0.05, and |log2FC| > 1 were assigned as differentially expressed.

### Analysis of biological significance of differential gene expression between treatments

To understand the biological significance of the observed differential gene expression profiles, lists of significant DEGs were subjected to GO (Young et al., 2010) and KEGG pathway enrichment analyses (Kanehisa, 2004). Both analyses were performed using the ClusterProfiler R package while correcting for gene set testing biases toward GO categories containing longer genes. Only the GO terms and KEGG pathways enriched with an adjusted *p-*value <0.05 (Benjamini–Hochberg correction for multiple testing) were considered significantly enriched by DEGs.

### Validation of gene expression with quantitative reverse-transcription PCR

To evaluate the reliability of the RNA-Seq data, two putative DEGs, omega-hydroxypalmitate *O*-feruloyl transferase (Soltu.Cru.03_4G014040; cutin, suberine and wax biosynthesis) and serine hydroxymethyltransferase (Soltu.Cru.05_3G016520; one-carbon metabolism), were reanalyzed using qRT-PCR. These genes were selected because of their statistical significance of repression or upregulation (*p*-value <0.05), as well as their potential role in PVY-induced tuber necrosis in potatoes.

Aliquots of total RNAs isolated for RNA-Seq were used for qRT-PCR experiments. For each sample, 1 μg of RNA was first processed to eliminate genomic DNA using a TURBO DNA-free™ kit (Invitrogen, Carlsbad, CA, USA) following the manufacturer’s instructions. First-strand cDNA synthesis was then performed using a commercial cDNA synthesis kit from Sangon Biotech (Shanghai, China). A 1:5 dilution of each cDNA was made in nuclease-free water (Thermo Fisher Scientific, Waltham, MA, USA) and stored at −20°C.

Coding sequences of the selected DEGs were obtained from the Potato Genomics Resource database (http://spuddb.uga.edu). Gene-specific primers for each DEG were then designed using the online PrimerQuest Tool (https://eu.idtdna.com/Primerquest/Home/Index) following default parameters and synthesized by Sigma-Aldrich (St. Louis, MO, USA). Prior to the qPCR assays, the annealing temperature and concentration for each primer were optimized using gradient PCR.

RT-qPCR was performed in 96-well plates with a C1000 Touch Thermal Cycler and CFX Real-Time PCR Detection System (Bio-Rad, Hercules, CA, USA). Each reaction (final volume of 10 µL) contained 1.0 μL of cDNA template, 5.0 μL of SsoAdvanced Universal SYBR Green Supermix (Bio-Rad, USA), 0.5 μL of each primer (10 μM), and 3.0 μL of nuclease-free water. The reaction profile had an initial pre-denaturation at 94°C for 3 minutes, followed by 30 cycles of denaturation at 94°C for 10 seconds, and a combined annealing and extension step at 58°C for 30 seconds. Two technical replicates of each of the three biological replicates along with a no template control were included in the qPCRs for each DEG.

The initial cycle threshold (Ct) or quantification cycle (Cq) data generated were then exported to an Excel file and used as input to compute 2^−Δδct^ values (or fold change estimates) in the expression of each DEG (Livak and Schmittgen, 2001), normalized to the eukaryotic elongation factor 1α (eEF1A), as previously used in similar experimental settings (Nicot et al., 2005; Goyer et al., 2015; Tang et al., 2017). Finally, the 2^−ΔΔCt^ data were then log-transformed and subjected to *t*-tests for statistical significance of the observed fold changes at a threshold of *p* < 0.05.

## Data availability statement

The data presented in the study are deposited in the GenBank BioSample repository, accession numbers SAMN40302237, SAMN40302238, SAMN40302239, SAMN40302240.

## Author contributions

RM: Writing – review & editing, Data curation, Formal analysis, Methodology, Validation, Writing – original draft. VS: Data curation, Formal analysis, Methodology, Writing – review & editing, Software, Supervision. HP: Supervision, Writing – review & editing, Conceptualization, Funding acquisition, Project administration, Resources.

## References

[B1] Amari K. Vazquez F. Heinlein M. (2012). Manipulation of plant host susceptibility: An emerging role for viral movement proteins? Front. Plant Sci. 3. doi: 10.3389/fpls.2012.00010 PMC3355624 22639637

[B2] Beczner L. Horváth J. Romhányi I. Förster H. (1984). Studies on the etiology of tuber necrotic ringspot disease in potato. Potato Res. 27, 339–352. doi: 10.1007/BF02357646

[B3] Ben-Dov C. Hartmann B. Lundgren J. Valcárcel J. (2008). Genome-wide analysis of alternative pre-mRNA splicing. J. Biol. Chem. 283, 1229–1233. doi: 10.1074/jbc.R700033200 18024428

[B4] Benedict C. A. McMoran D. W. Inglis D. A. Karasev A. V. (2015). Tuber symptoms associated with recombinant strains of potato virus Y in specialty potatoes under western washington growing conditions. Am. J. Potato Res. 92, 593–602. doi: 10.1007/s12230-015-9472-6

[B5] Brito C. Cabanes D. Sarmento Mesquita F. Sousa S. (2019). Mechanisms protecting host cells against bacterial pore-forming toxins. Cell. Mol. Life Sci. 76, 1319–1339. doi: 10.1007/s00018-018-2992-8 30591958 PMC6420883

[B6] Chung B. Y.-W. Miller W. A. Atkins J. F. Firth A. E. (2008). An overlapping essential gene in the Potyviridae. Proc. Natl. Acad. Sci. 105, 5897–5902. doi: 10.1073/pnas.0800468105 18408156 PMC2311343

[B7] Clarke P. G. H. (1990). Developmental cell death: Morphological diversity and multiple mechanisms. Anat. Embryology 181 (3), 195–213. doi: 10.1007/BF00174615 2186664

[B8] Conesa A. Madrigal P. Tarazona S. Gomez-Cabrero D. Cervera A. McPherson A.. (2016). A survey of best practices for RNA-seq data analysis. Genome Biol. 17, 13. doi: 10.1186/s13059-016-0881-8 26813401 PMC4728800

[B9] Divi U. K. Krishna P. (2009). Brassinosteroid: A biotechnological target for enhancing crop yield and stress tolerance. New Biotechnol. 26, 131–136. doi: 10.1016/j.nbt.2009.07.006 19631770

[B10] Dougherty W. G. Carrington J. C. (1988). Expression and function of potyviral gene products. Annu. Rev. Phytopathol. 26, 123–143. doi: 10.1146/annurev.py.26.090188.001011

[B11] Dupuis B. Bragard C. Schumpp O. (2019). Resistance of potato cultivars as a determinant factor of potato virus Y (PVY) epidemiology. Potato Res. 62, 123–138. doi: 10.1007/s11540-018-9401-4

[B12] Engelsdorf T. Horst R. J. Pröls R. Pröschel M. Dietz F. Hückelhoven R.. (2013). Reduced Carbohydrate Availability Enhances the Susceptibility of Arabidopsis toward *Colletotrichum higginsianum* . Plant Physiol. 162, 225–238. doi: 10.1104/pp.112.209676 23487433 PMC3641204

[B13] Filichkin S. A. Priest H. D. Givan S. A. Shen R. Bryant D. W. Fox S. E.. (2010). Genome-wide mapping of alternative splicing in *Arabidopsis thaliana* . Genome Res. 20, 45–58. doi: 10.1101/gr.093302.109 19858364 PMC2798830

[B14] Fink S. L. Cookson B. T. (2005). Apoptosis, pyroptosis, and necrosis: Mechanistic description of dead and dying eukaryotic cells. Infection Immun. 73, 1907–1916. doi: 10.1128/IAI.73.4.1907-1916.2005 PMC1087413 15784530

[B15] Gao M. Showalter A. M. (1999). Yariv reagent treatment induces programmed cell death in Arabidopsis cell cultures and implicates arabinogalactan protein involvement. Plant J. 19, 321–331. doi: 10.1046/j.1365-313X.1999.00544.x 10476079

[B16] Gao L. Tuo D. Shen W. Yan P. Li X. Zhou P. (2015). NIa-Pro of Papaya ringspot virus interacts with Carica papaya eukaryotic translation initiation factor 3 subunit G (CpeIF3G). Virus Genes 50 (1), 97–103. doi: 10.1007/s11262-014-1145-x 25416301

[B17] Garcia-Seco D. Chiapello M. Bracale M. Pesce C. Bagnaresi P. Dubois E.. (2017). Transcriptome and proteome analysis reveal new insight into proximal and distal responses of wheat to foliar infection by Xanthomonas translucens. Sci. Rep. 7, 10157. doi: 10.1038/s41598-017-10568-8 28860643 PMC5579275

[B18] Goyer A. Hamlin L. Crosslin J. M. Buchanan A. Chang J. H. (2015). RNA-Seq analysis of resistant and susceptible potato varieties during the early stages of potato virus Y infection. BMC Genomics 16, 472. doi: 10.1186/s12864-015-1666-2 26091899 PMC4475319

[B19] Gray S. De Boer S. Lorenzen J. Karasev A. Whitworth J. Nolte P.. (2010). *Potato virus Y*: An evolving concern for potato crops in the United States and Canada. Plant Dis. 94, 1384–1397. doi: 10.1094/PDIS-02-10-0124 30743397

[B20] Guo C. Sun L. Chen X. Zhang D. (2013). Oxidative stress, mitochondrial damage and neurodegenerative diseases. Neural Regen. Res. 8 (21), 2003–2014. doi: 10.3969/j.issn.1673-5374.2013.21.009 PMC4145906 25206509

[B21] Hagiwara-Komoda Y. Choi S. H. Sato M. Atsumi G. Abe J. Fukuda J.. (2016). Truncated yet functional viral protein produced via RNA polymerase slippage implies underestimated coding capacity of RNA viruses. Sci. Rep. 6, 21411. doi: 10.1038/srep21411 26898356 PMC4761962

[B22] Hardigan M. A. Laimbeer F. P. E. Newton L. Crisovan E. Hamilton J. P. Vaillancourt B.. (2017). Genome diversity of tuber-bearing *Solanum* uncovers complex evolutionary history and targets of domestication in the cultivated potato. Proc. Natl. Acad. Sci. 114 (46), E9999-E10008. doi: 10.1073/pnas.1714380114 PMC5699086 29087343

[B23] Harrington R. Katis N. Gibson R. W. (1986). Field assessment of the relative importance of different aphid species in the transmission of potato virus Y. Potato Res. 29, 67–76. doi: 10.1007/BF02361982

[B24] Hoopes G. Meng X. Hamilton J. P. Achakkagari S. R. De Alves Freitas Guesdes F. Bolger M. E.. (2022). Phased, chromosome-scale genome assemblies of tetraploid potato reveal a complex genome, transcriptome, and predicted proteome landscape underpinning genetic diversity. Mol. Plant 15, 520–536. doi: 10.1016/j.molp.2022.01.003 35026436

[B25] Isakov O. Modai S. Shomron N. (2011). Pathogen detection using short-RNA deep sequencing subtraction and assembly. Bioinformatics 27, 2027–2030. doi: 10.1093/bioinformatics/btr349 21666269 PMC3137223

[B26] Ivanov K. I. Eskelin K. Bašić M. De S. Lõhmus A. Varjosalo M.. (2016). Molecular insights into the function of the viral RNA silencing suppressor HCPro. Plant J. 85, 30–45. doi: 10.1111/tpj.13088 26611351

[B27] Kaiser A. Leistner E. (1990). Role of the entC gene in enterobactin and menaquinone biosynthesis in *Escherichia coli* . Arch. Biochem. Biophysics 276, 331–335. doi: 10.1016/0003-9861(90)90728-H 2154945

[B28] Kanehisa M. (2004). The KEGG resource for deciphering the genome. Nucleic Acids Res. 32, 277D–2 280. doi: 10.1093/nar/gkh063 PMC308797 14681412

[B29] Karasev A. V. Gray S. M. (2013). Continuous and emerging challenges of *potato virus Y* in potato. Annu. Rev. Phytopathol. 51, 571–586. doi: 10.1146/annurev-phyto-082712-102332 23915135

[B30] Karasev A. V. Nikolaeva O. V. Hu X. Sielaff Z. Whitworth J. Lorenzen J. H.. (2010). Serological properties of ordinary and necrotic isolates of potato virus Y: A case study of PVYN misidentification. Am. J. Potato Res. 87, 1–9. doi: 10.1007/s12230-009-9110-2

[B31] Knaggs A. R. (2003). The biosynthesis of shikimate metabolites. Natural Product Rep. 20, 119–136. doi: 10.1039/b100399m 12636087

[B32] Kroemer G. Galluzzi L. Vandenabeele P. Abrams J. Alnemri E. S. Baehrecke E. H.. (2009). Classification of cell death: Recommendations of the Nomenclature Committee on Cell Death 2009. Cell Death Differentiation 16, 3–11. doi: 10.1038/cdd.2008.150 18846107 PMC2744427

[B33] Lacomme C. Glais L. Bellstedt D. U. Dupuis B. Karasev A. V. Jacquot E. (Eds.) (2017). Potato virus Y: Biodiversity, pathogenicity, epidemiology and management (Cham, Switzerland: Springer International Publishing). doi: 10.1007/978-3-319-58860-5

[B34] Laine V. N. Gossmann T. I. Van Oers K. Visser M. E. Groenen M. A. M. (2019). Exploring the unmapped DNA and RNA reads in a songbird genome. BMC Genomics 20, 19. doi: 10.1186/s12864-018-5378-2 30621573 PMC6323668

[B35] Leist M. Jäättelä M. (2001). Four deaths and a funeral: From caspases to alternative mechanisms. Nat. Rev. Mol. Cell Biol. 2, 589–598. doi: 10.1038/35085008 11483992

[B36] Liao Y. Smyth G. K. Shi W. (2014). featureCounts: an efficient general purpose program for assigning sequence reads to genomic features. Bioinformatics. 30 (7), 923–930. doi: 10.1093/bioinformatics/btt656 24227677

[B37] Liu Z. Persson S. Sanchez-Rodriguez C. (2015). At the border: The plasma membrane-cell wall continuum. J. Exp. Bot. 66, 1553–1563. doi: 10.1093/jxb/erv019 25697794

[B38] Livak K. J. Schmittgen T. D. (2001). Analysis of relative gene expression data using real-time quantitative PCR and the 2–ΔΔCT method. Methods 25, 402–408. doi: 10.1006/meth.2001.1262 11846609

[B39] Manasseh R. Berim A. Kappagantu M. Moyo L. Gang D. R. Pappu H. R. (2023). Pathogen-triggered metabolic adjustments to potato virus Y infection in potato. Front. Plant Sci. 13. doi: 10.3389/fpls.2022.1031629 PMC9986423 36891131

[B40] Mandadi K. K. Scholthof K.-B. G. (2015). Genome-Wide Analysis of Alternative Splicing Landscapes Modulated during Plant-Virus Interactions in *Brachypodium distachyon* . Plant Cell 27, 71–85. doi: 10.1105/tpc.114.133991 25634987 PMC4330581

[B41] Martínez F. Daròs J.-A. (2014). Tobacco etch virus protein P1 traffics to the nucleolus and associates with the host 60S ribosomal subunits during infection. J Virol. 88 (18), 10725–10737. doi: 10.1128/JVI.00928-14 PMC4178839 24991017

[B42] Minina E. A. Filonova L. H. Sanchez-Vera V. Suarez M. F. Daniel G. Bozhkov P. V. (2013). Detection and measurement of necrosis in plants. Methods Mol. Biol. 1004, 229–248. doi: 10.1007/978-1-62703-383-1_17 23733581

[B43] Mishra J. Srivastava R. Trivedi P. K. Verma P. C. (2020). Effect of virus infection on the secondary metabolite production and phytohormone biosynthesis in plants. 3 Biotech. 10, 547. doi: 10.1007/s13205-020-02541-6 PMC7683645 33269181

[B44] Mortazavi A. Williams B. A. McCue K. Schaeffer L. Wold B. (2008). Mapping and quantifying mammalian transcriptomes by RNA-Seq. Nat. Methods 5, 621–628. doi: 10.1038/nmeth.1226 18516045 PMC13303166

[B45] Moyo L. Ramesh S. V. Kappagantu M. Mitter N. Sathuvalli V. Pappu H. R. (2017). The effects of potato virus Y-derived virus small interfering RNAs of three biologically distinct strains on potato (*Solanum tuberosum*) transcriptome. Virol. J. 14, 129. doi: 10.1186/s12985-017-0803-8 28716126 PMC5513076

[B46] Nagegowda D. A. (2010). Plant volatile terpenoid metabolism: Biosynthetic genes, transcriptional regulation and subcellular compartmentation. FEBS Lett. 584, 2965–2973. doi: 10.1016/j.febslet.2010.05.045 20553718

[B47] Nicot N. Hausman J.-F. Hoffmann L. Evers D. (2005). Housekeeping gene selection for real-time RT-PCR normalization in potato during biotic and abiotic stress. J. Exp. Bot. 56, 2907–2914. doi: 10.1093/jxb/eri285 16188960

[B48] Nilsen T. W. Graveley B. R. (2010). Expansion of the eukaryotic proteome by alternative splicing. Nature 463, 457–463. doi: 10.1038/nature08909 20110989 PMC3443858

[B49] Olspert A. Chung B. Y. Atkins J. F. Carr J. P. Firth A. E. (2015). Transcriptional slippage in the positive-sense RNA virus family *Potyviridae* . EMBO Rep. 16, 995–1004. doi: 10.15252/embr.201540509 26113364 PMC4552492

[B50] Oshlack A. Robinson M. D. Young M. D. (2010). From RNA-seq reads to differential expression results. Genome Biol. 11, 220. doi: 10.1186/gb-2010-11-12-220 21176179 PMC3046478

[B51] Pan Q. Shai O. Lee L. J. Frey B. J. Blencowe B. J. (2008). Deep surveying of alternative splicing complexity in the human transcriptome by high-throughput sequencing. Nat. Genet. 40, 1413–1415. doi: 10.1038/ng.259 18978789

[B52] Parekh S. Vieth B. Ziegenhain C. Enard W. Hellmann I. (2018). Strategies for quantitative RNA-seq analyses among closely related species. BioRxiv Preprint. doi: 10.1101/297408

[B53] Perring T. M. Gruenhagen N. M. Farrar C. A. (1999). Management of plant viral diseases through chemical control of insect vectors. Annu. Rev. Entomology 44, 457–481. doi: 10.1146/annurev.ento.44.1.457 15012379

[B54] Pertea M. Kim D. Pertea G. M. Leek J. T. Salzberg S. L. (2016). Transcript-level expression analysis of RNA-seq experiments with HISAT, StringTie and Ballgown. Nat. Protoc. 11, 1650–1667. doi: 10.1038/nprot.2016.095 27560171 PMC5032908

[B55] Piche L. M. Singh R. P. Nie X. Gudmestad N. C. (2004). Diversity among *potato virus Y* isolates obtained from potatoes grown in the United States. Phytopathology® 94, 1368–1375. doi: 10.1094/PHYTO.2004.94.12.1368 18943708

[B56] Quick R. A. Cimrhakl L. Mojtahedi H. Sathuvalli V. Feldman M. J. Brown C. R. (2020). Elimination of *Tobacco rattle virus* from viruliferous *Paratrichodorus allius* in greenhouse pot experiments through cultivation of castle russet. J. Nematol. 52, 1–10. doi: 10.21307/jofnem-2020-011 PMC7265893 32193908

[B57] Revers F. García J. A. (2015). Molecular biology of potyviruses. Adv. Virus Res. 92, 101–199. doi: 10.1016/bs.aivir.2014.11.006 25701887

[B58] Romancer M. L. Kerlan C. Nedellec M. (1994). Biological characterisation of various geographical isolates of potato virus Y inducing superficial necrosis on potato tubers. Plant Pathol. 43, 138–144. doi: 10.1111/j.1365-3059.1994.tb00563.x

[B59] Ross B. T. Zidack N. McDonald R. Flenniken M. L. (2022). Transcriptome and small RNA profiling of potato virus Y infected potato cultivars, including systemically infected russet burbank. Viruses 14, 523. doi: 10.3390/v14030523 35336930 PMC8952017

[B60] Samuels D. C. Han L. Li J. Quanghu S. Clark T. A. Shyr Y.. (2013). Finding the lost treasures in exome sequencing data. Trends Genet. 29, 593–599. doi: 10.1016/j.tig.2013.07.006 23972387 PMC3926691

[B61] Scholthof K.-B. G. Adkins S. Czosnek H. Palukaitis P. Jacquot E. Hohn T.. (2011). Top 10 plant viruses in molecular plant pathology: Top 10 plant viruses. Mol. Plant Pathol. 12, 938–954. doi: 10.1111/j.1364-3703.2011.00752.x 22017770 PMC6640423

[B62] Scott I. Logan D. C. (2008). Mitochondria and cell death pathways in plants: Actions speak louder than words. Plant Signaling Behav. 3, 475–477. doi: 10.4161/psb.3.7.5678 PMC2634434 19704490

[B63] Shrestha D. Wenninger E. J. Hutchinson P. J. S. Whitworth J. L. Mondal S. Eigenbrode S. D.. (2014). Interactions among potato genotypes, growth stages, virus strains, and inoculation methods in the potato virus Y and green peach aphid pathosystem. Environ. Entomology 43, 662–671. doi: 10.1603/EN13323 24690278

[B64] Song B. Jin L. Yang S. Bhadury P. S. (2010). Environment-Friendly Antiviral Agents for Plants (Berlin Heidelberg: Springer). doi: 10.1007/978-3-642-03692-7

[B65] Stein O. Granot D. (2019). An overview of sucrose synthases in plants. Front. Plant Sci. 10. doi: 10.3389/fpls.2019.00095 PMC6375876 30800137

[B66] Tang X. Zhang N. Si H. Calderón-Urrea A. (2017). Selection and validation of reference genes for RT-qPCR analysis in potato under abiotic stress. Plant Methods 13, 85. doi: 10.1186/s13007-017-0238-7 29075311 PMC5644265

[B67] Tholl D. (2006). Terpene synthases and the regulation, diversity and biological roles of terpene metabolism. Curr. Opin. Plant Biol. 9, 297–304. doi: 10.1016/j.pbi.2006.03.014 16600670

[B68] Trapnell C. Pachter L. Salzberg S. L. (2009). TopHat: Discovering splice junctions with RNA-Seq. Bioinformatics 25, 1105–1111. doi: 10.1093/bioinformatics/btp120 19289445 PMC2672628

[B69] Trump B. F. Berezesky I. K. (1996). The role of altered [Ca2+]i regulation in apoptosis, oncosis, and necrosis. Biochim. Biophys. Acta (BBA) - Mol. Cell Res. 1313, 173–178. doi: 10.1016/0167-4889(96)00086-9 8898851

[B70] Van Doorn W. G. Beers E. P. Dangl J. L. Franklin-Tong V. E. Gallois P. Hara-Nishimura I.. (2011). Morphological classification of plant cell deaths. Cell Death Differentiation 18, 1241–1246. doi: 10.1038/cdd.2011.36 21494263 PMC3172093

[B71] Wamonje F. O. Tungadi T. D. Murphy A. M. Pate A. E. Woodcock C. Caulfield J. C.. (2020). Three aphid-transmitted viruses encourage vector migration from infected common bean (Phaseolus vulgaris) plants through a combination of volatile and surface cues. Front. Plant Sci. 11. doi: 10.3389/fpls.2020.613772 PMC7767818 33381144

[B72] White A. C. Rogers A. Rees M. Osborne C. P. (2016). How can we make plants grow faster? A source–sink perspective on growth rate. J. Exp. Bot. 67, 31–45. doi: 10.1093/jxb/erv447 26466662

[B73] Whitham S. A. Yang C. Goodin M. M. (2006). Global impact: Elucidating plant responses to viral infection. Mol. Plant-Microbe Interactions® 19, 1207–1215. doi: 10.1094/MPMI-19-1207 17073303

[B74] Woolfson K. N. Esfandiari M. Bernards M. A. (2022). Suberin biosynthesis, assembly, and regulation. Plants 11, 555. doi: 10.3390/plants11040555 35214889 PMC8875741

[B75] Wu M. Ding X. Fu X. Lozano-Duran R. (2019). Transcriptional reprogramming caused by the geminivirus Tomato yellow leaf curl virus in local or systemic infections in Nicotiana benthamiana. BMC Genomics 20, 542. doi: 10.1186/s12864-019-5842-7 31272383 PMC6611054

[B76] Yadav D. K. Kumar S. Choi E.-H. Sharma P. Misra S. Kim M.-H. (2018). Insight into the molecular dynamic simulation studies of reactive oxygen species in native skin membrane. Front. Pharmacol. 9. doi: 10.3389/fphar.2018.00644 PMC6030362 29997501

[B77] Young M. D. Wakefield M. J. Smyth G. K. Oshlack A. (2010). Gene ontology analysis for RNA-seq: Accounting for selection bias. Genome Biol. 11, R14. doi: 10.1186/gb-2010-11-2-r14 20132535 PMC2872874

[B78] Yu M.-H. Zhao Z.-Z. He J.-X. (2018). Brassinosteroid signaling in plant–microbe interactions. Int. J. Mol. Sci. 19, 4091. doi: 10.3390/ijms19124091 30563020 PMC6320871

